# Histopathological Findings of Appendix Specimens in Quiescent Ulcerative Colitis: Correlations With Clinical Outcomes in the ACCURE Trial

**DOI:** 10.1002/ueg2.70177

**Published:** 2026-02-07

**Authors:** Eva Visser, Demy Danielsson, Lianne Heuthorst, Geert R. D'Haens, Willem A. Bemelman, Christianne J. Buskens, Aart Mookhoek

**Affiliations:** ^1^ Department of Surgery Amsterdam University Medical Centres University of Amsterdam Amsterdam the Netherlands; ^2^ Department of Gastroenterology and Hepatology Amsterdam University Medical Centres University of Amsterdam Amsterdam the Netherlands; ^3^ Institute of Tissue Medicine and Pathology University of Bern Bern Switzerland

## Abstract

**Background:**

The ACCURE trial demonstrated that appendicectomy reduces relapse rates within 1 year in patients with ulcerative colitis (UC) in remission. We aimed to explore appendiceal histopathology in quiescent UC and assess its association with postoperative relapse.

**Methods:**

Appendix specimens from Dutch participants in the ACCURE trial were reassessed by a blinded gastrointestinal pathologist using the Robarts Histopathology Index (RHI; range 0–33). Active appendiceal inflammation was defined as RHI > 3. Clinical data, preoperative endoscopic findings including peri‐appendiceal red patch (PARP), and outcomes were correlated with histopathological findings. Inter‐observer agreement between local and central scoring was assessed, along with relapse‐free survival in relation to RHI severity.

**Results:**

Of 65 patients, 49 (75.4%) maintained remission and 16 (24.6%) relapsed within one year. Active inflammation was present in 55.4% (36/65). Inter‐observer agreement was moderate (*κ* = 0.47, 95% CI 0.29–0.64, *p* < 0.001). Inflammation was more frequent in patients diagnosed at a younger age (median 28 vs. 34 years, *p* = 0.09), and greater in those with PARP (RHI 15.5 vs. 5.0, *p* = 0.005). Extensive epithelial neutrophil involvement (> 5% of crypts) was associated with higher relapse rates (44.4% vs. 18.0%, *p* = 0.05). Relapsing patients also had larger appendiceal diameters (median 9 vs. 7 mm, *p* = 0.03).

**Conclusion:**

Active appendiceal inflammation is prevalent in quiescent UC and showed a trend toward association with relapse risk. Although the benefit of appendicectomy in this group cannot be confirmed on these data alone, the finding might be clinically relevant as relapse rates are significantly reduced in the appendicectomy group.

## Key Summary

- Summarise the established knowledge on this subject
  ◦ The appendix is hypothesized to play a role in ulcerative colitis (UC) pathogenesis, with previous studies showing improved disease outcomes following appendicectomy.
  ◦ Active appendiceal inflammation has been found in UC patients regardless of endoscopic disease extent or activity.
  ◦ The ACCURE trial demonstrated reduced relapse rates after appendicectomy in patients with quiescent UC.
- What are the significant and/or new findings of this study?
  ◦ Active appendiceal inflammation (RHI > 3) was found in over half of the UC patients in clinical remission.
  ◦ Peri‐appendiceal red patch (PARP) and larger appendiceal diameter correlated with more severe histological inflammation.
  ◦ Presence of epithelial neutrophils (> 5% of crypts) was associated with a higher relapse risk post‐appendicectomy.
  ◦ Increasing RHI scores showed a non‐significant trend toward shorter time to relapse, suggesting a prognostic value of appendix histopathology in quiescent UC that requires confirmation in larger studies.

## 1 Introduction

Ulcerative colitis (UC) is a chronic inflammatory bowel disease characterized by histopathological features of both chronic and active inflammation limited to the colonic mucosa [1]. The hallmark of active inflammation is the presence of neutrophils in the mucosa, present in the lamina propria or epithelium (cryptitis, crypt abscess formation). Subsequent epithelial damage may lead to erosions and/or ulcerations. Chronicity is marked by structural changes such as crypt architectural distortion and Paneth cell metaplasia in the left colon and Paneth cell hyperplasia in the right colon [2]. Although histological healing is increasingly recognized as an important treatment target, achieving complete histological remission remains challenging and is not yet a formal treatment goal [3]. However, combined endoscopic and histological healing—referred to as histologic‐endoscopic mucosal improvement (HEMI) or remission (HEMR)—has been associated with improved treatment outcomes and is increasingly used as a clinical trial endpoint [4].

The role of the appendix in UC pathogenesis remains unclear, but a contribution has been hypothesized with recent trials reporting improved disease outcomes after appendicectomy [5, 6]. The appendix, an evolutionary remnant rich in gut‐associated lymphoid tissue (GALT), including abundant immune cells such as B and T lymphocytes and macrophages [7], is thought to modulate intestinal immune responses relevant to UC [8, 9]. Histopathological studies specifically examining the appendix in UC are limited, but existing data indicate that active appendiceal inflammation is present in approximately 50% of UC patients, irrespective of endoscopic disease activity or extent in the colon [7, 10, 11]. Notably, in active UC, active appendiceal inflammation has been associated with treatment response in these studies [7, 10, 11], further emphasizing the need for a deeper understanding of its potential role.

The ACCURE trial, the first randomized controlled trial evaluating appendicectomy as a therapeutic intervention in patients with quiescent UC, demonstrated a significantly lower relapse rate in those who underwent appendicectomy [12]. However, the primary trial publication focused solely on predefined clinical outcomes and did not report detailed appendiceal histopathology, leaving a gap in understanding its potential involvement in UC. Therefore, this study aims to further investigate the histopathology of the appendix in quiescent UC patients and examine its potential association with the clinical disease course in the ACCURE trial cohort.

## 2 Methods

### 2.1 Study Design and Population

This study was an investigator‐initiated histopathological analysis of appendix specimens obtained from participants in the Dutch cohort of the ACCURE trial (NTR2883), a randomized controlled trial evaluating the clinical effectiveness of appendicectomy in maintaining remission in UC patients. Detailed methodology and outcomes of the ACCURE trial have been published previously [12, 13, 14]. In short, the trial included UC patients in remission who had been treated for disease relapse within the preceding year but were not receiving advanced medical therapy (e.g., biologics or small molecules). Patients were randomized either to undergo laparoscopic appendicectomy in addition to continued maintenance therapy or to continue maintenance therapy alone.

For the present analysis, appendix specimens were collected from participants assigned to the appendicectomy arm within the Dutch cohort. Patients were excluded if they were diagnosed with Crohn's disease during follow‐up or if histopathological data of their resected appendix specimen was unavailable for blinded central assessment.

### 2.2 Data Collection and Variables

Baseline demographic and clinical characteristics were retrieved from the ACCURE trial database, including age at operation, age at UC diagnosis, sex (male or female), disease duration (from diagnosis to surgery), disease extent (proctitis [E1], left‐sided colitis [E2], or pancolitis [E3]), time from last exacerbation to appendicectomy, patient‐reported number of prior exacerbations, and partial Mayo score. Endoscopic parameters were retrieved from the endoscopy reports and included history of peri‐appendiceal red patch (PARP; if ever reported, and whether present on baseline endoscopy), and Mayo endoscopic score (MES; from baseline endoscopy). Appendiceal pathology reports from previously conducted evaluations at local participating centers were retrieved and specimen results were categorized into three groups: no inflammation (appendix sana), active inflammation, and fibrosis. Macroscopic appendiceal characteristics, including maximum diameter, were also collected from local pathology reports. Clinical follow‐up data included occurrence and timing of UC relapse, defined as confirmed relapse by either (1) endoscopy (Mayo subscore ≥ 2) or (2) elevated fecal calprotectin (> 150 μg/g) confirmed by centrally blinded review by a critical event committee. Data also included follow‐up MES and the date of the 12‐month follow‐up visit.

### 2.3 Histopathological Assessment

All available appendicectomy specimens were re‐evaluated by an independent expert gastrointestinal pathologist who was blinded to clinical data and outcomes. Tissue samples were formalin‐fixed, paraffin‐embedded and stained with hematoxylin and eosin for histological assessment. Histological scoring was performed using the Robarts Histopathology Index (RHI), a validated UC‐specific histological scoring system for assessing mucosal disease activity. The RHI assessed four characteristics of mucosal activity, each graded from 0 to 3 and multiplied by weighting factors: (1) Inflammatory infiltrate (x1); (2) Neutrophils in the lamina propria (x2); (3) Neutrophils in the epithelium (x3); and (4) Erosion or ulceration (x5). Total scores ranged from 0 to 33, with higher scores indicating greater inflammatory activity [15]. Active appendiceal inflammation was defined as an RHI > 3, consistent with established thresholds indicating the presence of histologic activity, and absence of histological appendiceal inflammation was defined as RHI ≤ 3 [16]. For further subgroup analyses, inflammation severity was categorized as remission (RHI ≤ 3), mild inflammation (RHI 4–10), moderate (RHI 11–20) and severe (RHI ≥ 21).

We additionally performed an assessment of chronic histological features of non‐fibrotic appendices. Chronicity markers included Paneth cell hyperplasia, crypt branching, crypt shortening, and crypt loss. The presence of any of these features was considered indicative of chronic mucosal damage. This evaluation was performed by the same blinded expert gastrointestinal pathologist to explore whether chronic changes accompanied active inflammation and whether these features differed between specimens with and without active inflammation (RHI > 3), and associations between chronicity markers and continuous RHI scores were explored using the Mann‐Whitney *U* test.

### 2.4 Clinical Outcome Assessment and Correlation Analysis

To evaluate its clinical relevance, histopathological features were correlated with baseline disease characteristics, preoperative endoscopic findings, and postoperative clinical outcomes. Comparisons were made between patients who maintained remission and those who experienced a relapse during follow‐up. Secondary outcomes included assessment of inter‐observer agreement between local pathologists and the central RHI scoring, and analysis of time‐to‐first‐relapse in relation to RHI severity categories.

### 2.5 Statistical Methods

Categorical variables were summarized using counts and percentages, and differences between groups were analyzed using the Chi‐square test or Fisher's exact test, as appropriate. Continuous variables were reported as medians with interquartile ranges (IQR) and compared using the Mann‐Whitney *U* test or Kruskal‐Wallis test, as appropriate. Inter‐observer agreement for histological active inflammation classification (local vs. expert pathologist) was evaluated using Cohen's kappa (*κ*), interpreted as follows: *κ* < 0.00 (poor agreement), 0.00–0.20 (slight), 0.21–0.40 (fair), 0.41–60 (moderate), 0.61–0.80 (substantial), and 0.81–1.00 (almost perfect) [17]. Correlations between baseline clinical characteristics and appendix RHI scores were assessed using Spearman's rank correlation (*ρ*) for continuous, and Mann‐Whitney U or Kruskal‐Wallis tests for categorical variables. The association between RHI scores and relapse was similarly analyzed with Mann‐Whitney *U* test. Kaplan‐Meier survival analysis with the log‐rank test was performed to compare time‐to‐relapse across RHI severity groups. Hazard ratios (HR) with 95% confidence intervals (CI), were calculated, considering relapse as the event of interest, while participants without relapse were censored at their last follow‐up visit. All tests were two‐sided, and a *p*‐value < 0.05 was considered statistically significant. Statistical analyses were conducted using SPSS version 28.0.1.1 (IBM, Armonk, NY, USA) or Stata version 17.0.0.

### 2.6 Ethics, Patients and Public Involvement

The study was approved by the Medical Ethics Review Committee of the Academic Medical Center on April, 12 2012 (NL37531.018.11) and adhered to the ethical guidelines and World Medical Association's Declaration of Helsinki. All patients provided written informed consent prior to any study‐related procedures.

## 3 Results

**FIGURE 1:**
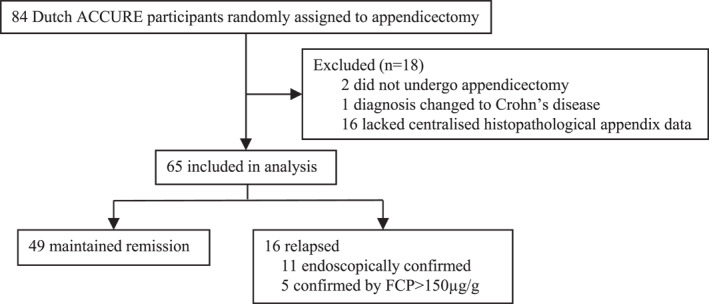
Flow diagram of ACCURE patients included in this analysis.

A total of 65 Dutch ACCURE participants randomly assigned to appendicectomy were included in this histopathology study (Figure 1). Centralized histological assessment was not available for 16 of the 84 Dutch appendicectomy specimens because the corresponding histopathology slides or tissue blocks could not be retrieved or delivered in time for central review. Among the included patients, 49 (75.4%) maintained remission, while 16 (24.6%) had experienced a relapse within one year. Of these relapses, 11 were confirmed by endoscopy, and 5 were confirmed by FCP levels exceeding 150 μg/g. Baseline demographic and clinical characteristics are summarized in Table 1. Overall, patients had a median age at appendicectomy of 40 years (IQR, 33–49) and a median disease duration of 5.2 years (IQR, 2.4–11.9). Disease extent was evenly distributed: proctitis (E1) in 36.9% (24/65), left‐sided colitis (E2) in 33.8% (22/65), and pancolitis (E3) in 29.2% (19/65). A history of PARP was previously documented in 16 patients (24.6%), with 4 cases during trial baseline endoscopy.

**TABLE 1:** Demographic and clinical baseline characteristics of the cohort (*n* = 65).

Characteristic		Active appendiceal inflammation^a^		
	Cohort (*n* = 65)	Yes *N* = 36	No *n* = 29	*p*‐value
Age at operation, years	40 (33–49)	39 (32–49)	41 (34–56)	0.26
Age at diagnosis, years	31 (25–39)	28 (24–35)	34 (26–46)	0.09
Sex, female	36 (55.4%)	20 (55.6%)	16 (55.2%)	0.98
Disease duration, years	5.2 (2.4–11.9)	5.2 (1.6–13.0)	5.2 (2.8–11.1)	0.91
Disease extent				0.67
Proctitis (E1)	24 (36.9%)	15 (41.7%)	9 (31.0%)	
Left‐sided (E2)	22 (33.8%)	11 (30.6%)	11 (37.9%)	
Pancolitis (E3)	19 (29.2%)	10 (27.8%)	9 (31.0%)	
Time since last exacerbation, months	7.8 (5.1–11.5)	7.1 (4.9–11.6)	8.1 (5.3–11.5)	0.88
Number of prior exacerbations (patient‐reported)	5 (3–10)	5 (3–10)	5 (2–7)	0.62
Peri‐appendiceal red patch history	16 (24.6%)	11 (30.6%)	5 (17.2%)	0.09
At baseline endoscopy	4 (6.2%)	4 (23.5%)	0 (−)	0.08
Mayo endoscopic score at baseline				0.98
0	26 (48.1%)	14 (48.3%)	12 (48.0%)	
1	28 (51.9%)	15 (51.7%)	13 (52.0%)	
Partial mayo score at baseline = 0	48 (73.8%)	26 (72.2%)	22 (75.9%)	0.74
Maximum diameter appendix, mm^b^	7 (5–10)	8 (6–10.1)	6 (5–7.8)	**0.02**

*Note:* Data are median (IQR) or number (%). Missing data for time since last exacerbation: 2 patients; 9 for number of exacerbations; 11 for endoscopy (confirmed by fecal calprotectin level < 150 μg/g according to protocol). Bold values indicate statistical significance (*p* < 0.05).

^a^ Active appendiceal inflammation was defined as RHI > 3.

^b^ 16 not reported.

### 3.1 Histopathological Findings of the Appendix

**FIGURE 2:**
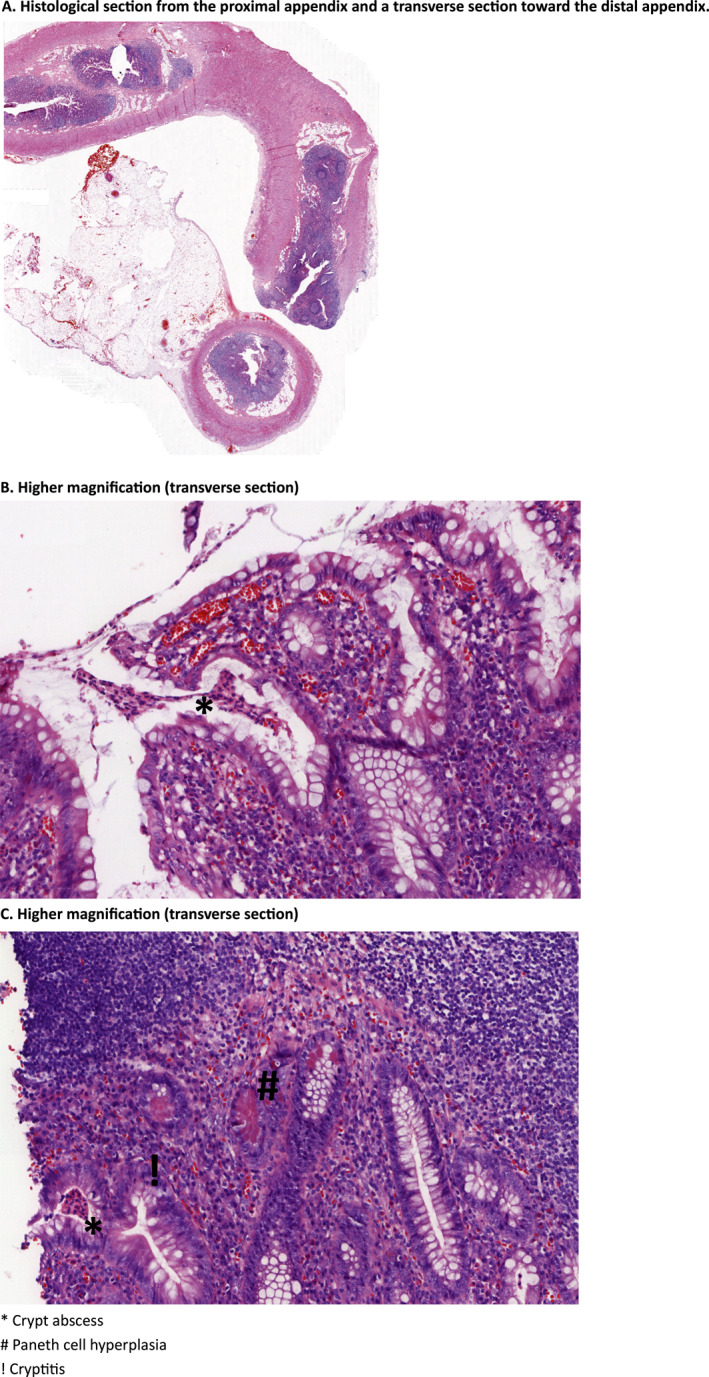
Appendix with severe active inflammation.

Central reading showed a median RHI of 6 (IQR, 0–13) in the overall cohort. Active inflammation was present in 36 of 65 (55.4%) of the appendices, while 29 (44.6%) had no inflammation. Of these without inflammation, 9 appendices (13.8%) showed complete fibrous obliteration of the appendiceal lumen and therefore no RHI could be scored. Excluding fibrotic appendices, the median RHI was 7 (IQR, 1–15). The highest RHI observed was 31 where the appendix showed severe active inflammation (Figure 2). Detailed histological findings (Table 2) of the 56 non‐fibrotic appendices indicated that most patients demonstrated a mild (41% [23/56]) or moderate (32% [18/56]) increase in chronic inflammatory infiltrates. Neutrophilic infiltration was also prevalent, with 59% (33/56) showing lamina propria neutrophils and 66% (37/56) showing epithelial neutrophils. Erosions or ulcerations were present in 27% (15/56) of the evaluated specimens, although severe erosion or ulceration were uncommon (1.8% [1/56]).

**TABLE 2:** Histopathological characteristics of the resected appendix specimen (*N* = 65).

Characeristics		Number/total number [n/N] (%)
Total fibrotic obliteration		9 (13.8%)
RHI, median (IQR)^a^		6 (0–13)
Chronic inflammatory infiltrate (x1)		
(0)	No increase	12/56 (21.4%)
(1)	Mild increase	23/56 (41.1%)
(2)	Moderate increase	18/56 (32.1%)
(3)	Marked increase	3/56 (5.4%)
Lamina propria neutrophils (x2)		
(0)	No increase	23/56 (41.1%)
(1)	Mild but unequivocal increase	14/56 (25.0%)
(2)	Moderate increase	19/56 (33.9%)
(3)	Marked increase	0/56 (−)
Epithelial neutrophils (x3)		
(0)	None	19/56 (33.9%)
(1)	< 5% crypts involved	19/56 (33.9%)
(2)	< 50% crypts involved	12/56 (21.4%)
(3)	> 50% crypts involved	6/56 (9.2%)
Erosion or ulceration (x5)		
(0)	No erosion, ulceration or granulation tissue	41/56 (73.2%)
(1)	Recovering epithelium + adjacent inflammation	8/56 (14.3%)
(2)	Unequivocal erosion	6/56 (10.7%)
(3)	Ulcer or granulation tissue	1/56 (1.8%)
Active inflammation (RHI > 3)^b^		35 (54.7%)
Reported by local pathologist		
Appendiceal inflammation		
No (appendix sana)		21 (32.3%)
Active inflammation		28 (43.1%)
Fibrosis		16 (24.6%)

^a^ Median RHI without fibrotic appendices: 7 (IQR, 1–15).

^b^ 9 patients with TFO are considered as absence of appendiceal inflammation (RHI = 0).

Chronicity markers were common in the evaluated appendices. The presence of any chronicity feature was associated with significantly higher RHI scores (absence of chronicity: median RHI 1.0 [IQR, 0.0–8.0] vs. presence of chronicity: median RHI 7.5 [IQR, 4.5–16.0]; *p* = 0.039). Overall, chronicity markers were observed in 84.8% (28/33) of assessable appendices. These features were substantially more frequent in appendices with active inflammation compared with those without (95.7% [22/23] vs. 60.0% [6/10], *p* = 0.021).

Local pathology reports classified 32.3% of specimens as appendix sana, 43.1% as active inflammation, and 24.6% as fibrosis. Inter‐observer agreement between local pathologist and centralized RHI scoring by the expert IBD pathologist was moderate (*κ* = 0.467, 95% CI 0.29 to 0.64, *p* < 0.001). Most discrepancies were found in the scoring of appendix sana by the local pathologist, whereas the central reading scored active inflammation (*n* = 10). The RHI in these 10 cases ranged from 4 to 15.

### 3.2 Association Between Baseline Characteristics and Active Appendiceal Inflammation

Baseline demographic and clinical characteristics were compared between patients with (RHI > 3) and without (RHI ≤ 3) active appendiceal inflammation. No significant associations were observed in age at appendicectomy, sex, disease duration, disease extent, time since last exacerbation, number of prior exacerbations, Mayo endoscopic score, or partial Mayo score (Table 1). Patients with active appendiceal inflammation showed a trend toward a younger median age at UC diagnosis compared to those without inflammation (28 vs. 34 years, *p* = 0.09). Similarly, there was a trend toward a numerically higher prevalence of PARP compared to those without it (30.6% vs. 17.2%, *p* = 0.09), with the presence of PARP significantly associated with higher median RHI scores (15.5 [IQR, 12.8 to 22.8] vs. 5.0 [IQR, 0.0 to 9.5], *p* = 0.005). Correlation analyses between continuous RHI scores and baseline characteristics yielded similar results.

### 3.3 Association Between Appendix Histopathology and Disease Course

**FIGURE 3:**
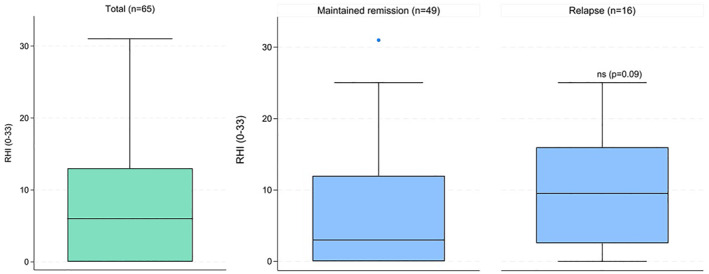
Appendix histopathology and disease course. RHI, Robarts Histopathology Index.

The relationship between appendiceal histopathology and clinical disease course post‐appendicectomy was further explored. The median maximum diameter was significantly greater in patients who relapsed post‐surgery compared with those who remained in remission (9 mm [IQR, 7–10.3] vs. 7 mm [IQR, 5–8] *p* = 0.03). Additionally, higher RHI scores showed a trend toward correlation with the maximum diameter (*ρ* = 0.254, *p* = 0.08). Patients who experienced a relapse demonstrated numerically higher RHI scores compared with those maintaining remission (9.5 [IQR, 1.8 to 16.5] vs. 3 [IQR, 0.0 to 12.0], *p* = 0.09; Figure 3). Extensive epithelial neutrophil involvement (> 5% of crypts) showed a trend toward higher relapse rates (44.4% vs. 18.0%, *p* = 0.05). Among the 36 patients with active appendiceal inflammation, those who relapsed were younger at the time of UC diagnosis (25.0 years [IQR, 21.5 to 29.5] vs. 32.0 [IQR, 26.3 to 38.0], *p* = 0.04) and had a longer disease duration (9.0 years [IQR, 5.2 to 30.9] vs. 4.6 [IQR, 1.1 to 8.6], *p* = 0.04). Among the 29 patients without appendiceal inflammation, these differences were not observed (*p* = 0.44 and *p* = 0.78, respectively).

**FIGURE 4:**
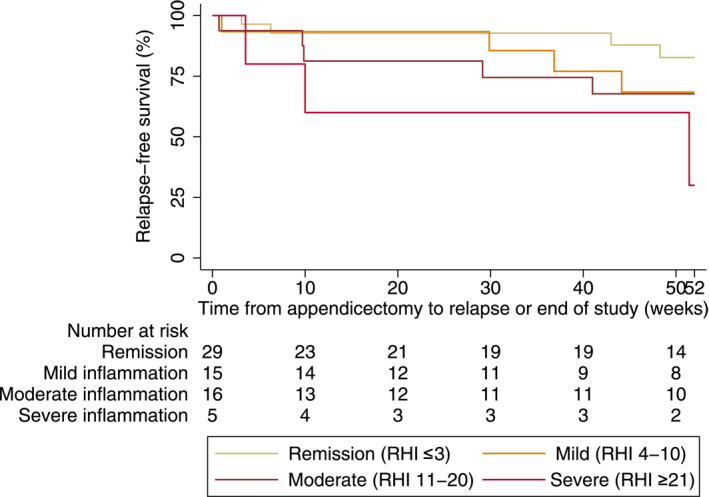
Kaplan‐Meier survival subgroups.

Kaplan‐Meier survival analysis demonstrated a clear trend toward shorter time‐to‐relapse with increasing appendiceal histological severity (Figure 4). At 12 months, relapse‐free survival was highest in patients without appendiceal inflammation (82%) and progressively decreased across those with mild (RHI 4–10; 71%), moderate (RHI 11–20; 68%), and severe inflammation (RHI ≥ 21; 36%). Although not statistically significant, patients with active appendiceal inflammation showed a trend toward a higher hazard of relapse compared with those in histological remission (HR 2.7; 95% CI 0.76 to 10.13, *p* = 0.12).

## 4 Discussion

This study provides the first detailed histopathological analyses of appendix specimens in quiescent UC directly linked to clinical outcomes following appendicectomy, conducted within a randomized controlled trial (ACCURE). We demonstrated that active appendiceal inflammation was common in quiescent UC and was present in over half of the patients (55%). Notably, a history of PARP was significantly associated with more severe appendiceal inflammation (higher RHI scores). Although not statistically significant, we observed trends toward a younger age at UC diagnosis (*p* = 0.09) and a greater maximum appendiceal diameter (*p* = 0.08) in patients with active appendiceal inflammation compared with those without. Furthermore, active appendiceal inflammation was numerically associated with a 2.7‐fold increased hazard of relapse post‐appendicectomy (*p* = 0.12) Overall, these findings indicate a potential trend toward an association between appendiceal histopathology and the clinical course of UC. Nevertheless, no statistically significant relationships were identified in this cohort, and the study was not powered to detect differences. Thus, these results require cautious interpretation.

Our results align with previous observational studies reporting a high prevalence of active appendiceal inflammation in UC patients, regardless of disease activity or extent [7, 10, 11]. Neutrophilic infiltration, notably both in the lamina propria and epithelium of the appendix, was frequent (59% and 66%, respectively). Importantly, moderate neutrophilic infiltration in the lamina propria of the appendix emerged as a potential histopathological marker associated with relapse, consistent with prior research identifying neutrophilic activity as an indicator of colonic mucosal inflammation and predictor of relapse [15, 18]. Extensive epithelial neutrophil involvement (> 5% crypts involved) significantly correlated with higher relapse rates, reinforcing the clinical relevance of neutrophilic infiltration patterns as a prognostic marker. These findings in quiescent UC support the theory that the appendix may serve as a persistent immune‐priming site in UC, potentially contributing to ongoing subclinical inflammation and relapse despite apparent mucosal healing in the colon [8, 19, 20, 21]. Among patients with inflamed appendices, those who relapsed had a longer disease duration compared with those who maintained remission. Given the overall reduction in relapse rates post‐appendicectomy, this observation that non‐relapsing patients with inflammation had a shorter disease duration supports the hypothesis that appendicectomy might be more effective when performed earlier in the disease course.

The finding that chronicity markers were associated with higher RHI scores supports the interpretation that these inflammatory patterns represent UC‐related appendiceal involvement. Unlike isolated acute appendicitis, which typically lacks chronic architectural alterations, the coexistence of chronic mucosal changes and neutrophilic activity is characteristic of IBD‐associated appendiceal inflammation [10]. These combined features strengthen the hypothesis that the appendix may serve as a site of persistent immune activation in UC rather than reflecting incidental acute inflammation.

Appendiceal fibrosis, by contrast, was associated with a numerically lower relapse rate compared to non‐fibrotic appendices (13% vs. 28%), suggesting that it may represent a post‐chronic active inflammatory state that has transitioned to an immunologically inactive phase. However, these possibilities cannot be differentiated with histology assessment at a single time point, and fibrosis may also reflect physiological age‐related changes unrelated to disease activity.

The association between PARP and higher RHI scores (PARP+: RHI 15.5 vs. PARP–: RHI 5.0, *p* = 0.005) aligns with previous suggestions that peri‐appendiceal inflammatory changes may indicate active local immune processes influencing the clinical course of UC [7, 9]. Although PARP has been correlated with a more aggressive disease course [22], its utility as a clinical indicator for underlying active appendiceal inflammation appears limited. Only a minority of patients (31%) with a histologically confirmed active appendiceal inflammation exhibited a visible PARP during colonoscopy. As such, PARP may primarily identify a small subset of patients with pronounced local inflammation, and additional diagnostic approaches modalities may be needed to reliably detect appendiceal involvement.

Macroscopic appendiceal characteristics observed in this study further support this need. Patients who relapsed had a significantly larger maximum appendiceal diameter compared with those who remained in remission (9 vs. 7 mm, *p* = 0.03). Although the absolute difference was small, appendiceal diameters remain of interest because they may reflect underlying inflammatory or luminal processes. This suggests that the appendiceal diameter might serve as a clinically accessible preoperative predictor of relapse risk. This interpretation is supported by prior studies in the general population, where increased appendiceal diameter measured by ultrasound is an established diagnostic criterion for acute appendicitis [23]. Although exploratory in our cohort, non‐invasive modalities such as intestinal ultrasound could therefore offer valuable support in patient stratification, warranting further research in the context of UC.

The strength of this study includes its prospective design within a rigorous randomized trial setting and blinded centralized histological assessment. A key limitation of this study is the inability to directly assess the therapeutic impact of appendicectomy, as appendix specimens from the control (non‐appendicectomised) group were unavailable. This prohibits any conclusions on causality, and limits the interpretation of the clinical relevance of the effect of appendicectomy on recurrence in patients with active appendiceal inflammation. Consequently, the identification of preoperative indicators—such as PARP or increased appendiceal diameters—becomes critical for inferring treatment effect in future research. Early findings from the ACCURE trial suggest that these markers may help identify patients most likely to benefit from appendicectomy. Hypothetically, if the procedure reduces relapse risk by 20% and this benefit is concentrated in patients with PARP or larger appendiceal diameter, such markers would enable targeted patient selection. Another limitation is that the study was not powered to detect differences in histopathological subgroups, limiting statistical significance.

In conclusion, our findings demonstrate that active appendiceal inflammation is frequently present in patients with quiescent UC, with several non‐significant trends toward higher relapse risk. Whether this is the group most benefitting from appendicectomy remains to be determined. Future larger‐scale studies are warranted to further investigate the prognostic value of active appendiceal inflammation and evaluate surrogate markers—such as PARP and intestinal ultrasound—as non‐invasive indicators of appendiceal involvement.

## Author Contributions

E.V. contributed to the conceptualization, formal analysis, investigation, validation, and original writing of the manuscript. D.D. participated in the investigation. L.H., G.R.D’.H., W.A.B. and A.M. were involved in the investigation and provided resources, as well as contributed to the review and editing of the manuscript. C.J.B. contributed to the conceptualization, investigation and supervision, and participated in the review and editing process. All authors reviewed the work critically for important intellectual content, approved the final version to be published, and agreed to be accountable for all aspects of the work.

## Funding

The ACCURE trial was funded by Nuts‐Ohra (FNO 1202‐008) and Dr. Falk Pharma in the Netherlands. The funding sources supported the design and implementation of the ACCURE trial but were not involved in this analysis or manuscript preparation.

## Ethics Statement

The study was approved by the Medical Ethics Review Committee of the Academic Medical Center on April, 12 2012 (NL37531.018.11) and adhered to the ethical guidelines and World Medical Association's Declaration of Helsinki.

## Consent

All patients provided written informed consent prior to any study‐related procedures.

## Conflicts of Interest

GRD’H reports receiving grants from Pfizer, Takeda, AbbVie, Eli Lilly, BMS, and Alimentiv; consulting fees from AbbVie, Agomab, Alimentiv, AstraZeneca, Bristol‐Myers Squibb, Boehringer Ingelheim, Celltrion, Eli Lilly, Exeliom Biosciences, Index Pharmaceuticals, GlaxoSmithKline, Pfizer, Johnson & Johnson, Polpharma, Procise Diagnostics, Prometheus Laboratories, Prometheus Biosciences, and Ventyx; payment for lectures from AbbVie, Arena, Boehringer Ingelheim, Bristol‐Myers Squibb, Celltrion, Eli Lilly, Johnson & Johnson, Pfizer, and Takeda; and travel support from Eli Lilly and Pfizer; and participation on data safety monitoring boards or advisory boards for Galapagos, AstraZeneca, and Seres Health. WAB reports received speaker fees from Applied Medical and Johnson & Johnson. CJB reports received speaker fees from Takeda, Janssen, and Tillotts Pharma. EV DD, LH, AM declared no conflict of interest.

## Data Availability

The data that support the findings of this study are available on request from the corresponding author. The data are not publicly available due to privacy or ethical restrictions.
